# Ascending Aortic Wall Fibroelastoma in an Elderly Woman with Dyspnea

**DOI:** 10.14797/mdcvj.1081

**Published:** 2022-06-28

**Authors:** Nausharwan Butt, Asma Gulab, Ji Hyun Yook, Iman Abdulameer Faraj Alsaray, Lovely Chhabra

**Affiliations:** 1 Einstein Medical Center, Philadelphia, Pennsylvania, US; 2 Shanghai Medical College of Fudan University, Shanghai, CN; 3 Al Mustansiriyah Medical College, Baghdad, IQ; 4 Mid-Hudson Regional Hospital, Poughkeepsie, New York, US

**Keywords:** ascending aorta, aortic fibroblastoma, dyspnea, anticoagulation, surgery

## Abstract

Aortic fibroelastoma is an uncommon pathology that is often found incidentally on routine cardiac imaging. The use of multimodality imaging including computerized tomography and magnetic resonance imaging has led to discovery of further distinguishing features of these lesions that may allow improved differentiation from aortic thrombi. Although most are located on cardiac valves, nonvalvular fibroelastomas have been seen on occasion. Optimal diagnosis and management of incidental aortic fibroelastomas remains debated. We describe a case of nonvalvular aortic fibroelastoma and review current diagnostic and management approaches.

## Introduction

Fibroelastomas (FE) are described as small benign tumors incorporating avascular, papillary, and pedunculated tissue surrounded by a single covering of endothelium.^1^ Cardiac FE are relatively rare clinical entities. These pathologic tissues tend to be found incidentally on routine clinical imaging, with most patients asymptomatic at the time of diagnosis.^1,2^ However, individuals with FE on occasion may be predisposed to an increased risk of embolization with significant morbidity and mortality, hence diagnosis and management of this clinical condition remains of noteworthy interest. Whereas most cases tend to be attached to the aortic and mitral valves, nonvalvular locations have been described in a handful of case studies.^3,4,5,6^ FE may share common clinical features to thrombi, vegetations, or other tumors, so differentiating these etiologies can be a diagnostic challenge. Whereas isolated aortic thrombi may resolve with conservative anticoagulation, FE likely require surgical excision to reduce the risk of systemic embolization.^3,7^

This report describes a large ascending aortic wall fibroelastoma and provides a case-based review of the pathophysiology and treatment approach of this clinical condition.

## Case Description

A 77-year-old woman with a medical history of hypertension, type 2 diabetes mellitus, tobacco use, chronic obstructive pulmonary disease, and recent diagnosis of paroxysmal atrial fibrillation presented for an outpatient cardiac evaluation with complaints of gradually progressive dyspnea on exertion and chest heaviness. The patient was initiated on anticoagulation, given apixaban 5 mg twice daily for a week for paroxysmal atrial fibrillation (AF) prior to the clinical visit.

**Figure 1:**
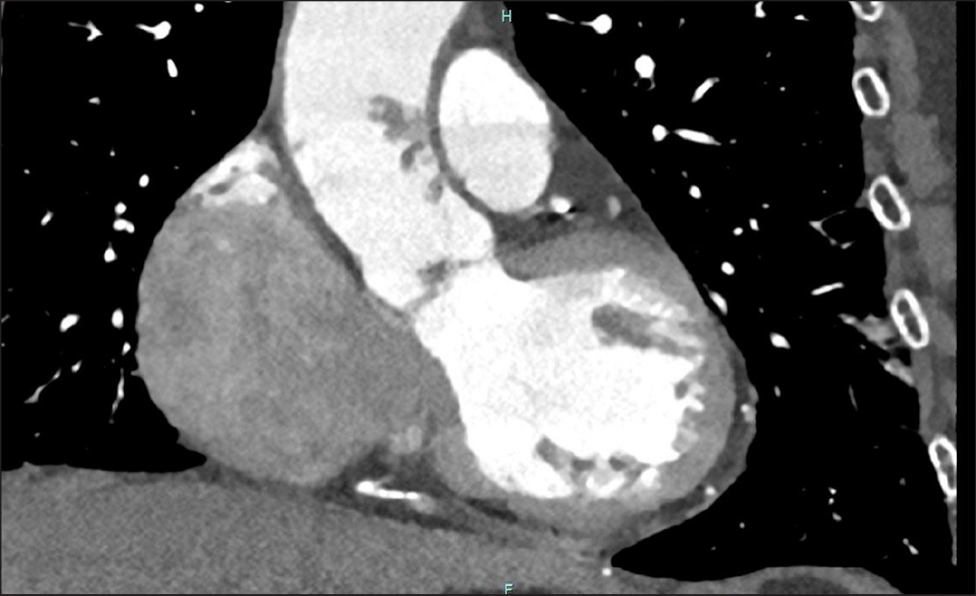
Longitudinal axial computed tomographic angiography image revealing a pedunculated aortic mass in the ascending aorta.

**Figure 2:**
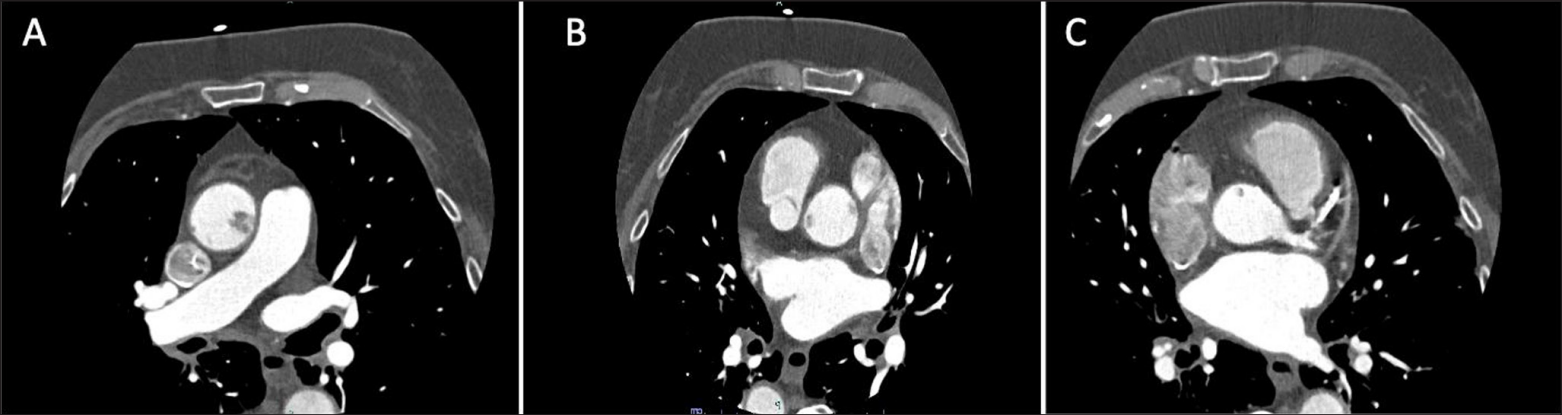
**(A–C)** Transverse axial images of coronary computed tomographic angiography at different axial levels demonstrate coronary contrast filling defects in the ascending aorta consistent with suspected aortic thrombus (finally determined to be primary aortic fibroelastoma by histopathology).

The patient was hemodynamically stable on presentation. The electrocardiogram revealed rate-controlled AF without signs of acute ischemia or infarction. Physical examination was largely unremarkable except for mild wheezing. Given significant risk factors for coronary artery disease, coronary computerized tomography angiogram (CCTA) and transthoracic echocardiogram (TTE) were obtained. The TTE was largely unremarkable except for mild left atrial enlargement. CCTA revealed extensive multivessel coronary artery calcification, including severe proximal left anterior descending artery (LAD) and proximal right coronary artery (RCA) stenosis in addition to an incidental finding of a significant area of hypoattenuation in the ascending thoracic aorta, near the coronary ostia, which was radiographically most likely consistent with a pedunculated aortic thrombus (Figures 1, 2 A-C).

Cardiac catheterization with coronary angiography was delayed given the proximity of the lesion to the coronary ostia and the potential risk for clot dislodgement and embolism. The patient was continued on full-dose anticoagulation with apixaban for another 2 weeks before subsequently repeating a magnetic resonance angiogram (MRA), which demonstrated no clear evidence of the aortic lesion previously noted on the CCTA. The hypercoagulable panel, including factor V Leiden, lupus anticoagulant, homocysteine levels, and a methylenetetrahydrofolate reductase homozygous gene mutation panel were unremarkable.

**Figure 3:**
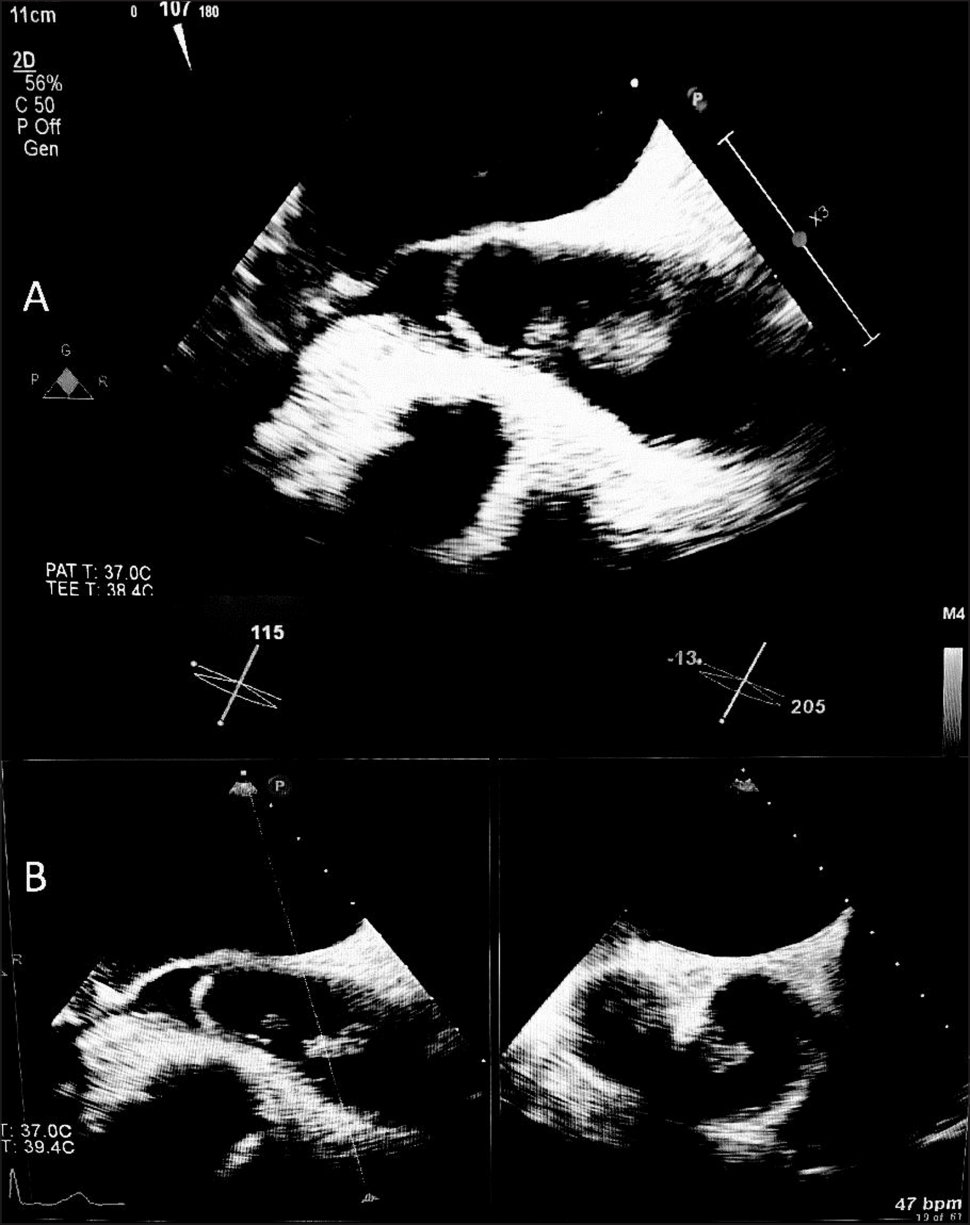
**(A)** Transesophageal view demonstrating a mobile pedunculated aortic mass in the ascending aorta, superior to the aortic valve. **(B)** The same aortic mass is visualized across an x-plane (perpendicular plane) view of the transesophageal echocardiogram.

Assuming resolution of a possible aortic wall thrombus, a diagnostic coronary angiography was performed, which confirmed the presence of severe two-vessel coronary artery disease involving the proximal LAD and proximal RCA. Subsequently, the patient was referred for an elective surgical revascularization. Anticoagulation was continued, and she was evaluated by hematology. A repeat thorax/abdomen/pelvis CTA was requested by hematology, which again showed multiple filling defects within the ascending thoracic aorta. The largest one measured approximately 9 mm and was best visualized along the left side of the ascending thoracic aorta with a portion in proximity to the left coronary ostium. An intraoperative transesophageal echocardiogram (TEE) during elective surgical revascularization scheduled 1 month later still demonstrated a mobile mass in the ascending aorta, which was concerning for a grade 4 mobile atheroma, or thrombus, or an aortic tumor (Figure 3 A, B). She underwent two-vessel bypass surgery with left internal mammary artery to LAD and saphenous vein graft to distal right coronary artery (RCA) utilizing a y-shaped graft and hemiarch replacement using a Hemashield aortic graft (Getinge).

**Figure 4:**
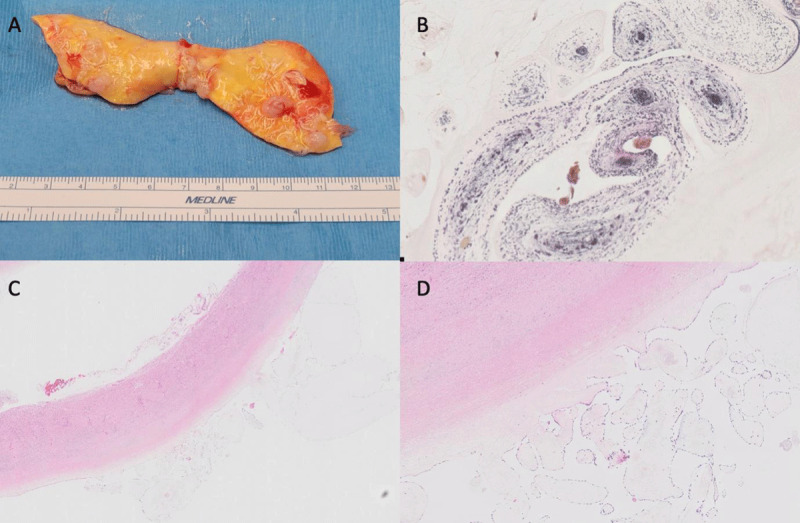
**(A)** Gross pathology of aortic fibroelastomas: the specimen presented is a part of the ascending aorta. The intimal surface demonstrates multiple papillary areas in different locations, with pink tan to yellowish discoloration with some mild focal calcification consistent with aortic fibroelastoma. **(B)** Verhoeff-Van Gieson/EVG stain highlights the elastic cores on histopathology images confirming the diagnosis of fibroelastoma. **(C, D)** Histopathology slides on different magnification scale demonstrate avascular fibroelastic tissue lined by a single layer of cells with multiple branching points, consistent with papillary fibroelastoma.

The removed ascending aorta was sent for further evaluation in three separate pieces measuring 1.5 × 0.8 × 0.2 cm to 11 × 3.8 × 0.3 cm. Separate specimen analysis of friable granular papillary intimal surface quantified as 0.2 × 0.2 × 0.1 cm to 1.1 × 0.8 × 0.2 cm with pink tan to yellowish discoloration and some focal areas of calcification (Figure 4 A). Although the mass morphologically had the appearance of a sea anemone similar to a myxoma, the histopathology with CD34 and Verhoeff–Van Gieson/EVG stains highlighted the elastic cores confirming the diagnosis of aortic wall fibroelastoma (Figure 4 B–D).

On outpatient clinical follow-up, the patient demonstrated a significant interval improvement in her cardiac symptoms.

## Discussion

Our case study describes a patient who presented with progressive dyspnea who was initially treated for incidental primary aortic thrombus but was later found to have an extremely rare incidence of nonvalvular aortic wall papillary FE. In a retrospective cohort analysis by Gowda et al., usual locations of papillary FE were the aortic (36%), mitral (29%), tricuspid (11%), and pulmonary (7%) valves.^8^ FE located on the aortic wall are particularly unusual and, to the best of our knowledge, have only been described three times previously.^4,5,6^

Whereas FE is uncommon, it is a significant source of noncardiogenic emboli with a high rate of complications; thus, timely diagnosis and management is essential to reduce adverse events. It is believed that the incidence of papillary FE was previously underestimated until recent advancements in imaging modalities.^3^ Our case in particular underscores the importance of multimodality imaging in establishing a successful clinical diagnosis. Whereas TEE helps provide initial characteristics regarding FE, including location and mobility, CTA and MRA can supply additional detail, including high-resolution 3-dimensional imaging, which allows further characterization of the aortic mass.^9^

Although CTA and TEE allowed the visualization of the aortic FE in our patient, subsequent MRA did not allow a clear visualization of the pathology. While MRA usually has a good sensitivity and specificity for diagnosis of aortic lesions, it has lower spatial resolution compared to CTA. Its contrast resolution also may be dependent on other factors, including the tissue-pathology interface, and such factors may have played a role in inadequate diagnosing of the aortic pathology earlier in our case. In addition to these factors, technical factors such as a suboptimal acquisition technique are possible. This case further underscores the importance of multimodality cardiovascular imaging in challenging cases.

The ideal management approach continues to be debated and is not well-established in the literature, especially for incidental FEs.^10^ In patients presenting with embolic strokes who have acceptable operative risk, excision of the lesions at highly experienced surgical centers has been the standard of care. In addition, the excision of papillary FE through minimally invasive approaches has been described. In patients who have high surgical risk, use of anticoagulation should be considered to prevent emboli from superimposed thrombi on the tumor.^1,3,6,7,8,9,10^

In our patient, symptoms of dyspnea were most likely due to the presence of underlying two-vessel significant coronary artery disease. However, gross surgical evaluation revealed the mass to be larger in size than first attributed by CT while also in proximity to the coronary ostium. Thus, clinical symptoms may have been exacerbated by intermittent obstruction of the coronary flow, caused by the papillary FE itself at the ostial level, as also evidenced by the small contrast filling defect on axial CT images at the levels across and in close proximity to the left coronary ostium (Figure 2 B, C).

Surgical intervention within our case also may have prevented a potential catastrophic embolic coronary blockade. This possible sequalae has been reported previously by Rolf et al. in a case of acute myocardial infarction from coronary embolization of an aortic FE of the thoracic ascending aorta.^4^

Our case study highlights an extremely rare yet very important diagnosis that should be considered in the differential diagnosis of patients with a primary aortic mass.

## Conclusion

Diagnosis and management of cardiovascular tumors such as primary aortic FE remain challenging. Use of multimodality imaging can aid in the diagnosis and clinical management of these patients and also may prevent unwanted adverse events. In patients with symptomatic tumors, surgical resection ideally should be performed at cardiac centers with special expertise. However, management of asymptomatic or incidentally diagnosed papillary FE remains controversial, and shared clinical decision with use of multidisciplinary evaluation is highly recommended.
